# Enhanced Low-temperature Electro-optical Kerr Effect of Stable Cubic Soft Superstructure Enabled by Fluorinated Polymer Stabilization

**DOI:** 10.1038/s41598-017-11041-2

**Published:** 2017-09-04

**Authors:** Xiao Li, Wei-Qiang Yang, Cong-Long Yuan, Zhen Liu, Kang Zhou, Xiao-Qian Wang, Dong Shen, Zhi-gang Zheng

**Affiliations:** 1 Physics Department, East China University of Science and Technology, Shanghai, 200237 China; 2 School of Materials Science and Engineering, East China University of Science and Technology, Shanghai, 200237 China

## Abstract

An enhanced electro-optical Kerr effect of the stable self-organized cubic blue phase liquid crystal superstructure at a relatively low temperature down to −50 °C was achieved through a judiciously designed fluorinated polymer stabilization. The fluorinated sample exhibited not only a rather stable cubic structure, but the promoted electro-optical performances of low driving voltage, weak hysteresis and high contrast ratio at such a low-temperature, which were much distinct from the common non-fluorinated polymer stabilized blue phase liquid crystal without conspicuous low-temperature Kerr response behaviours. Kerr constant, which reflects the obviousness of Kerr effect, of the fluorinated sample at −50 °C indicated a spectacular enhancement of two orders of magnitude in contrast to the commonly material, thereby corroborating the high efficiency of polymer fluorination in enhancing low-temperature Kerr effect. Such an enhancement of Kerr effect was probably resulted from the decreasing of interfacial anchoring between liquid crystal and fluorinated polymer network. The fluorinated polymer stabilization not only ensures the stability of self-organized cubic structure of blue phase, but overcomes the challenge and bottleneck problem of low-temperature inapplicability of common blue phase liquid crystal and paves a brilliant and broad way for relevant materials to abundant perspective applications at low temperature.

## Introduction

Self-organization in soft matter systems, constructing a three-dimensional (3D) cubic superstructure with a lattice constant in the scale of several hundreds of nanometers, reveals a charming and fantastic molecular behaviour in the framework of *bottom-up* nanofabrication^1–3^. Blue phase liquid crystal (BPLC), commonly existing in a high-chirality liquid crystal system, is such a typical self-organized soft system, which can be further categorized into three sub-phases in accordance with liquid crystal (LC) arrangement—BPIII exhibiting a fluent fog-like texture formed by isotropic symmetrical double twisted cylinder (DTC) of chiral LCs, BPII and BPI characterized by colourful platelets textures owing to their exotic 3D cubic lattice superstructure stacked by DTCs, *i.e*., simple cubic for BPII, while body-centered cubic for BPI^4–8^. Such arrangement enables BPLC unique properties of optical isotropy, sub-millisecond response time and selective reflection^9–11^, which are desirable and of great practical significance in perspective applications not only limited in displays but others beyond. However, the prominent drawback—thermal instability—of BPLC, displaying a narrow temperature range of less than 5 °C, promotes sustained researches to develop new stable BPLC materials in the past decade^9, 12–15^, especially the polymer stabilized BPLC (PSBPLC)^16, 17^, which has been considered as one of the most potential candidates adopted in displays^18–21^ and versatile photonic devices, such as polarization-independent and fast responsive phase grating^22, 23^, microlens array^24^ and photonic band edge laser^25, 26^.

Tremendous explorations and efficient endeavours, contraposing to enhance Kerr effect and suppress the hysteresis of BPLC, were contributed in the last decade by judiciously designing the shape of electrodes, such as the protrusion electrode^19^, corrugated electrode^27^ and the partitioned wall electrode^28^, and by strategically replacing the common used in-plane switching (IPS) driving mode with vertical field switching (VFS)^29^. Furthermore, the great efforts on the improvements of materials properties were sufficiently impressive. A hysteresis-free BPLC with low driving voltage was achieved at a temperature higher than room temperature (~50 °C) by doping a tiny amount of zinc sulphide (ZnS) nanoparticles^30^, due to the high dipole moment of such nanoscale dopant; on the other aspect, the mono-functional acrylate monomers with a long alkyl chain, dodecyl acrylate, were mixed to reduce the interfacial anchoring between LCs and polymer network in PSBPLC system^31^. A fast response and low driving voltage BPLC, exhibiting indistinguishable gamma shift and wide viewing angle, was specifically developed for field-sequential-colour display by the combination of the optimizations on materials and structures of device^18^.

Herein, we report an efficient strategy to enhance Kerr effect of PSBPLC at the low temperature enabled by a judiciously designed fluorination of polymer network. The optimized material exhibited a conspicuous electric-field induced Kerr effect at −50 °C, achieving a remarkable enlarged Kerr constant which was approximately two orders of magnitude larger with respect to the aforementioned prior result^32^. Although the polymer fluorination was previously employed in polymer-LC material system to promote the room-temperature performances of relevant devices^33^, low-temperature-applicable PSBPLC with desirable EO Kerr performance was still a challenging issue. Therefore, such an interesting Kerr effect enhancement of PSBPLC at a relatively low temperature down to −50 °C realized through polymer fluorination opens a window for BPLC and relevant other LCs to be adopted in the low-temperature environment, which is sufficiently significant in practical applications not only limited in out-door displays, but the aerospace optical systems.

## Results and Discussions

### Fluorinated polymer stabilization of BPLC

**Figure 1:**
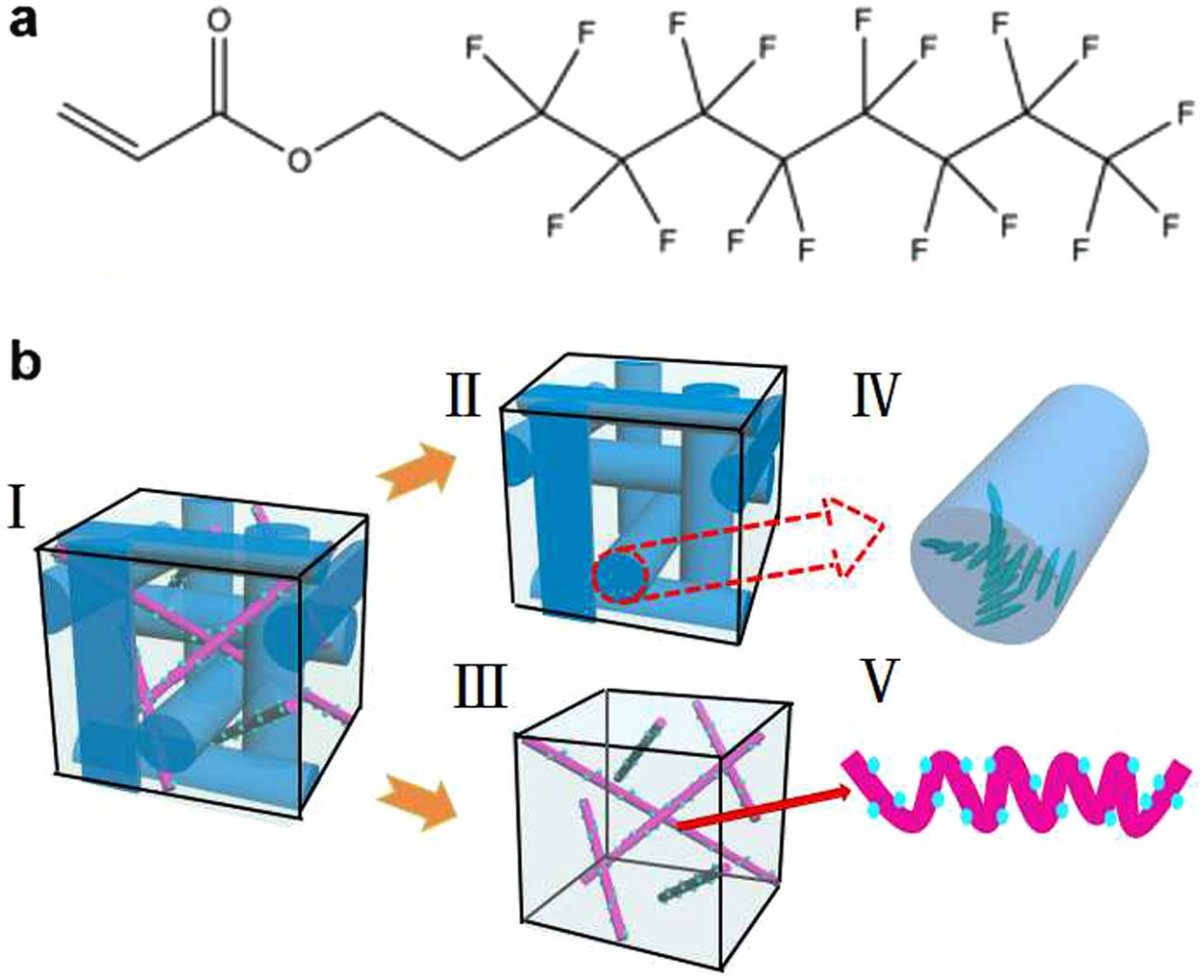
(**a**) Chemical structure of PFDA; (**b**) Schematic diagram of the structure of the fluorinated polymer stabilized blue phase liquid crystal (I) and blue phase liquid crystal (II). Blue phase liquid crystal commonly coexists with the isotropic aligned defect (III) and the double twisted cylinder (IV). LC arrangement in defects is stabilized by the fluorinated polymer (V, cyan balls in figure V denote the fluorine).

**Figure 2:**
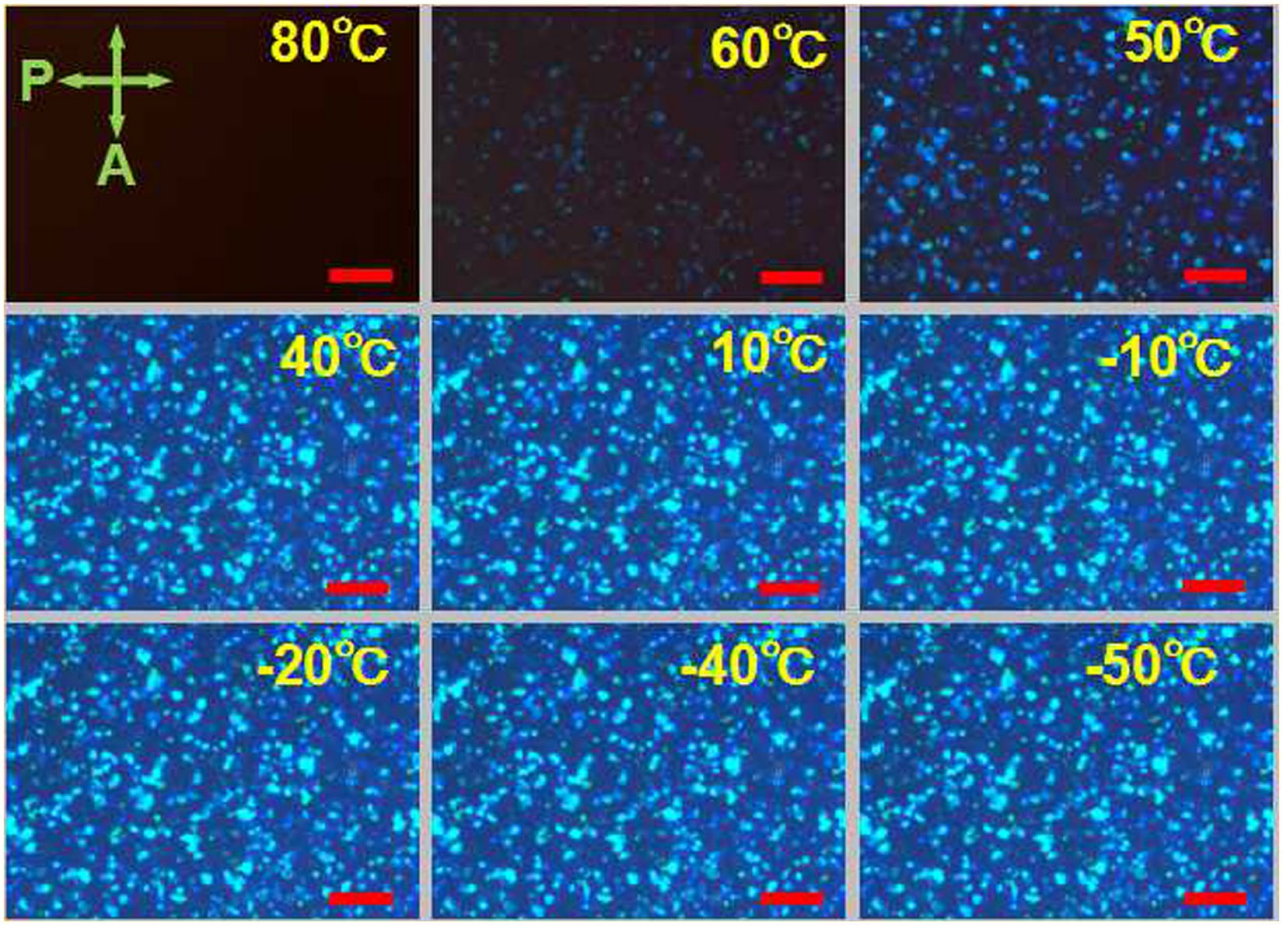
Optical textures of the fluorinated PSBPLC sample SF6 (i.e., 6 wt% of PFDA monomers were mixed into the system before polymer stabilization) observed using reflection mode of POM with crossed polarizers. The corresponding temperature is labelled at the top right corner of every panel. Scale bar: 100 μm.

As aforementioned, BP is a typical frustrated LC phase having a coexistence of the double-twisted arranged LC, i.e., DTC (blue cylinders in Fig. 1b -IV), and the isotropic aligned LC, i.e., the defects. The defect lines, playing a crucial role like the “scaffolds” (schematically depicted as the pink slim rods inserted into the interspaces between the DTCs, Fig. 1b-I) to fix such cubic structure, are stabilized by photo-polymerizing the fluorinated monomers, therefore forming the fluorinated PSBPLC with the majority of fluorine atoms (cyan balls in Fig. 1b-V) distribute on the interface between DTCs and defects due to the strong electronegativity of fluorine. At the room temperature, the voltage-dependent-transmission (VT) performance of the samples presented an abrupt decline of the driving voltage for about 41%, from 95 V to 56 V, as the content of 1H,1H,2H,2H-Perfluorodecyl Acrylate (PFDA) was increased to 4 wt%; and followed by a mild decreasing to 50 V with a continuous increasing of PFDA to 6 wt% (Fig. S1, Supplementary). Further enhancement of fluorination (*i.e*., the content of PFDA reached 8 wt%) resulted in undesired phase transition from BP to chiral nematic (N*) phase instead of a corresponding expected improvement of the driving voltage during photo polymerization, owing to the promotion of molecular motion on one hand and a slight reduction of the average functionality of the monomers on the other hand. The BP of the sample was maintained in a wide temperature range spanning more than 110 °C with the enhancement of fluorination. Texture evolutions of the sample doped with 6 wt% PFDA during the cooling from isotropic state indicated that BP appeared at ~60 °C and presented a typical texture of dark blue platelets at 50 °C; the reflection colour of platelets gradually transformed to bluish and bright cyan with a continued cooling to 40 °C and retained at such state even the temperature reached −50 °C, which was almost the limitation of the hot stage used herein (Fig. 2). The upper-limitation of BP slightly decreased with the increasing of PFDA, 60 °C for the system doped 6 wt% PFDA and 66 °C for the sample without fluorination, resulting from the weakening of the interfacial anchoring effect after the fluorination. BP texture of the sample, whether be fluorinated or not, can be maintained even the temperature decreased down to the limitation of hot stage, −50 °C.

### The enhancement of Kerr effect by fluorination

**Figure 3:**
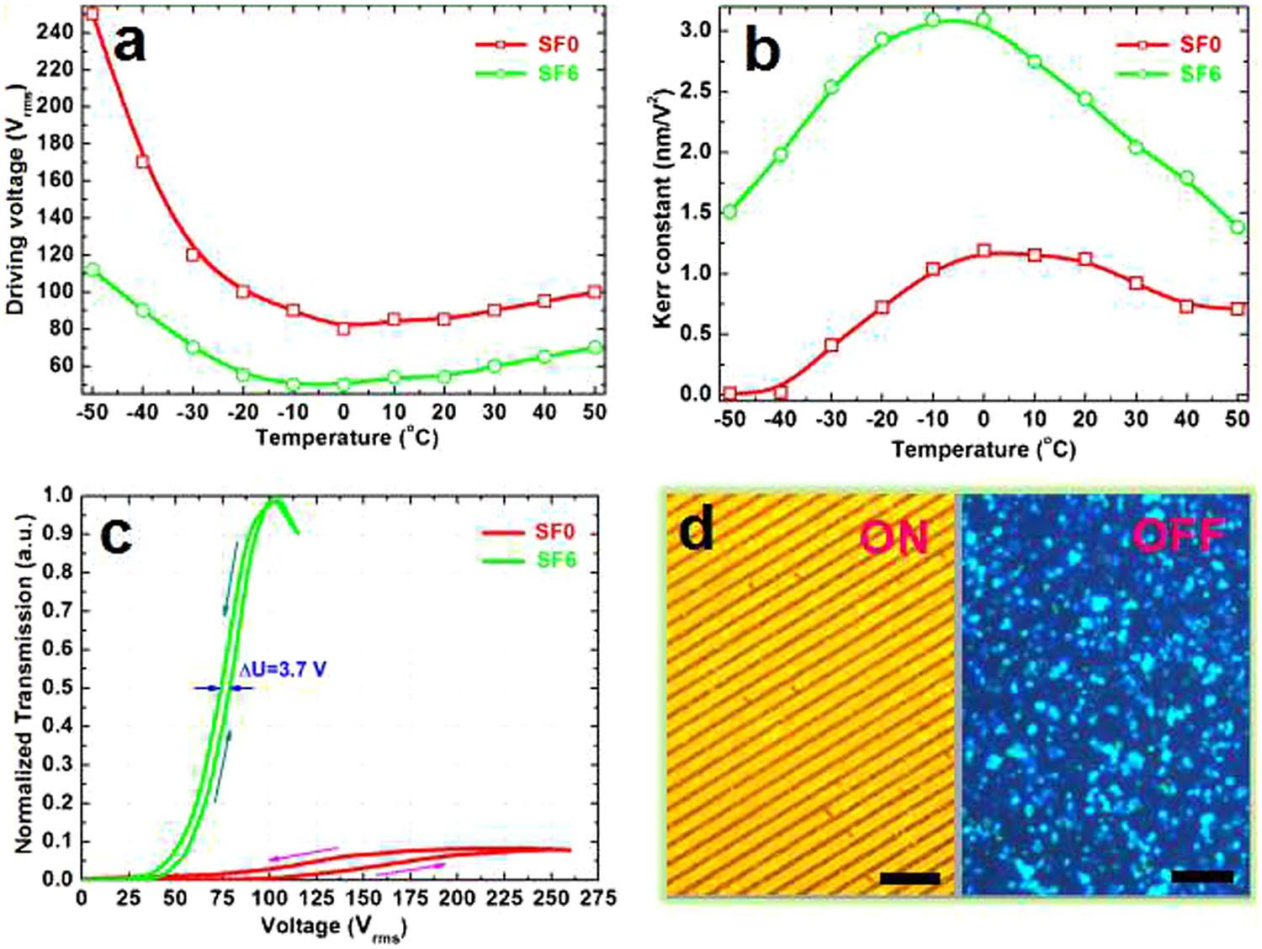
The enhanced Kerr effect of fluorinated PSBPLC sample. (**a**) Temperature dependent driving voltage of the non-fluorinated sample, SF0, and the fluorinated sample, SF6 during a cooling from 50 °C to −50 °C; the corresponding Kerr constant is shown in (**b**). (**c**) Electro-optical hysteresis behaviour of the samples SF0 and SF6 tested at −50 °C. Hysteresis (ΔU) herein was defined as the voltage-differences at 50% transmittance of the loop (*i.e*., width of the loop at 50% transmittance). Herein, the tested transmissions were normalized by the maximum transmission of the sample SF6. (**d**) The ON and OFF state of the fluorinated sample SF6 at −50 °C after undergoing 100 cycles of voltage-applied and removed. Voltage: 115 V, scale bar: 100 μm.

The comparison of EO performances between the samples containing 0 wt% and 6 wt% PFDA (denoted as SF0 and SF6 respectively in the following presentations) during the cooling process confirmed a significant Kerr effect enhancement of SF6 resulted from fluorination. The temperature dependent driving voltage (Fig. 3a) indicated that the driving voltage of SF6 decreased for about 30~40% compared with that of SF0 at the temperature above 0 °C; moreover, such decreasing was gradually enlarged with a further cooling to −50 °C. When the temperature was −50 °C, the driving voltage of SF6 reduced more than a half, 55.2%, compared with the sample without fluorination, *i.e*., SF0. Interestingly, the driving voltages of the two samples presented a similar tendency with the variation of temperature, *i.e*., a slight decreasing with the cooling from 50 °C to 0 °C owing to a better arrangement of LCs at a lower temperature; and followed by a remarkable rising with a continued cooling to −50 °C because of the comparably strong interfacial anchoring between polymer and LCs at the temperature below 0 °C. However, in the region of low temperature, the driving voltage of SF0 rose more abruptly in contrast with SF6, which was probably ascribed to polymer fluorination. The corresponding Kerr constant of SF6, reflecting the obviousness of Kerr effect, ascended significantly during the cooling stage from 50 °C to −10 °C and followed by a rapid reduction till −50 °C. However, as for the non-fluorinated SF0, the variation of Kerr constant was comparatively milder (Fig. 3b). The tested Kerr constant of SF6 reached the maximum in the temperature range of −10~0 °C, which was approximately three times of the peak value of SF0 obtained in the range of 0~10 °C. As the temperature reduced down to the limitation of hot stage, −50 °C, Kerr effect of SF0 almost disappeared, with a corresponding rather low Kerr constant of less than 0.01 nm/V^2^; in contrast, the sample SF6 still exhibited a conspicuous Kerr effect, with a relatively high Kerr constant, which was two orders of magnitude higher compared with the sample SF0 and other common PSBPLC (Fig. 3b).

**Figure 4:**
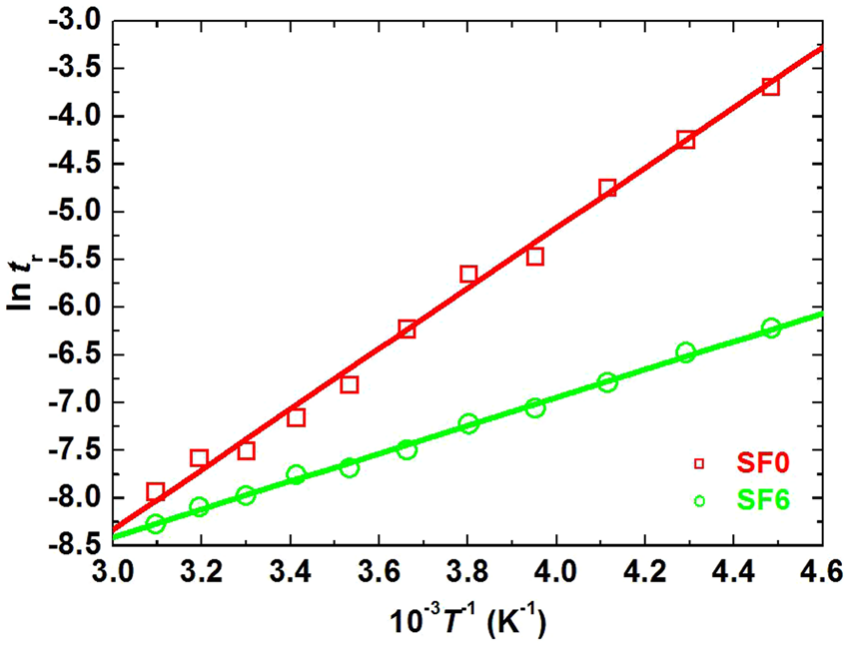
The tested response time during the cooling stage from 50 °C to −50 °C of the non-fluorinated sample SF0 (red open squares) and fluorinated sample SF6 (green open circles). The corresponding data were fitted in accordance with the linear relationship, ln(*t*_r_) = −(*E*_b_ *T* ^−1^)/R + ln(*t*_0r_), deduced from Arrhenius equation. Therefore, the energetic barrier *E*_b_ can be calculated through the slope, −(*E*_b_/R), of fitted line. The response time was tested by applying a driving voltage of the sample at the corresponding temperature.

To further corroborate the enhancement of Kerr effect at low temperature after the fluorination, the aforementioned samples, SF0 and SF6, were cooled to −50 °C to measure their VT curves (Fig. 3c). A satisfying tunability of fluorinated SF6, similar as that tested in room temperature (Fig. S2 Supplementary), was remained, displaying a relatively high contrast ratio of approximately 1000 and a slight EO-hysteresis (*i.e*., ΔU, Fig. 3c) of 3.7 V; contrarily, a very low maximum transmission of SF0, corresponding to a contrast ratio of less than 10, with serious EO-hysteresis issue, indicated a severely weakened Kerr effect of such a common non-fluorinated PSBPLC. As expected, the driving voltage of SF6 still reduced about 60% compared to SF0, although LC viscosity was inevitably risen at such a low working temperature. The response behaviour at −50 °C was significantly improved via fluorination, revealing one order of magnitude shortening of the fluorinated SF6 compared with the non-fluorinated SF0 (Fig. 4). Furthermore, a 115-V voltage was applied and removed on the sample SF6 at −50 °C for more than 100 cycles, both a bright voltage-applied state (ON state) and a typical BP state (OFF state) remained unchanged during the cycles (Fig. 3d), thereby reflecting a reliable operability of the fluorinated sample. Such a remarkable electro-optical Kerr effect reinforces the adaptation of BPLC and other LC based displays at a low temperature, improves the image blurring caused by the low electric-field induced birefringence, and thereby solves the bottleneck problem of the related devices which are commonly inapplicable in the low-temperature environment, such as the polar region where a device heater is requisite for a normal work of device. Furthermore, such material is promising to be applied as an optical load of spacecraft. In general, a low weight and low-temperature applicability are two requirements of the space payload, considering the economization of power source of the craft. Thus, such material will be a powerful candidate without doubt.

### Analysis of interfacial effects

A sample having an enhanced Kerr effect commonly exhibits a lower energetic barrier of molecular realignment (*i.e*., a low driving voltage), which was explored in accordance with Arrhenius equation, *t*_r_ = *t*_r0_exp(−*E*_b_/R*T*), where, *t*_r_ is the EO rise time of PSBPLC; *T* is the temperature; R is known as a constant; while *t*_r0_ is the rise time when the *T* reaches infinity; therefore, the energetic barrier *E*_b_ is obtained by fitting and calculating the slope of a linear dependency between ln(*t*_r_) and *T* ^−1^. Accordingly, *E*_b_ of the fluorinated SF6 and the non-fluorinated SF0 were confirmed to be −12.2 kJ/mol and −26.3 kJ/mol, respectively (Fig. 4; SF6: green line connecting green open circles; SF0: red line connecting red open squares). Herein, the value of such energetic barrier is in proportion to the elastic constant, the viscosity of LCs as well as the interfacial anchoring of polymer network in PSBPLC; while the minus means the resistant effect of the energetic barrier to the external driving energy. In the case that the contents of LCs and the chiral dopant in SF6 and SF0 were invariable, the lower energetic barrier of SF6 was quite probably resulted from a weaker interfacial anchoring of fluorinated polymer, which was further confirmed by a gradually increased contact angle of LCs on fluorinated polymer surface (Fig. S3 Supplementary) and the decreased driving voltage (Fig. S1 Supplementary) with the enhancement of fluorination. Furthermore, the interfacial interaction energy between LCs and polymer, calculated through the molecular dynamics (MD) method (see details in Supplementary Information), theoretically clarified the weakening of interfacial anchoring, which indicated that the interaction energy was reduced for about 16% after polymer fluorination.

## Conclusions

In conclusion, an enhanced Kerr effect of the cubic self-organized PSBPLC superstructure at the low temperature was achieved through a judiciously designed polymer fluorination. The fluorinated PSBPLC presented a wide temperature range of more than 110 °C, spanning from a BP entrance point lower than −50 °C to a clearing point higher than 60 °C. As the temperature was cooled down to −50 °C, the PSBPLC treated by polymer fluorination exhibited a significant enhanced Kerr effect with adequate prominent advantages on EO performances compared with the common PSBPLC—a relatively high contrast ratio of almost 1000, which was two orders of magnitude higher than the common PSBPLC; a low driving voltage, which was less than a half of the common PSBPLC; and a weak EO hysteresis of only 3.7 V. The Kerr constant of the fluorinated PSBPLC was thus improved remarkably, which was two orders of magnitude higher relative to the common PSBPLC with an extremely low Kerr constant of less than 0.01 nm/V^2^. The enhancement of Kerr effect was probably resulted from the reduction of interfacial anchoring between the fluorinated polymer and LCs on accordance with the experimental results and the theoretical simulation. Such enhanced Kerr effect of PSBPLC at a low temperature down to −50 °C realized by polymer fluorination paves a brilliant way for abundant perspective applications of BPLC and other relatives in the environment of low temperature, such as polar-region and aerospace adaptive systems.

## Methods

### Materials preparation

The BPLC host, possessing a BP range from 55.0 °C to 52.5 °C, was prepared by mixing a commercial nematic LC denoted as TEB300 (supported by Slichem Co., Ltd., China) and a common chiral agent R5011 (supported by HCCH, China) with a weight ratio of 96.4:3.6. For implementing polymer stabilization of such cubic arrangement, a certain amount of acrylate-based monomers accompanied by trace amounts of photoinitiator (Irgacure 184, provided by BASF) were accurately doped into the aforementioned host. Herein, the monomers were composed by a kind of reactive mesogen, RM257 (supported by Merck), and the flexible 2-ethylhexyl acrylate (2-EHA), mixed with a weight ratio of 2:1. The fluorination was achieved by partially replacing the common acrylate monomer with a judiciously selected fluorinated acrylate monomer with a long fluorocarbon chain, PFDA (herein, supported by Sigma-Aldrich, Fig. 1a). The weight concentration of PFDA in the whole prepared mixture for PSBPLC was modulated from 2 wt% to 8 wt% by a step of 2 wt%. Herein, monomer concentration was optimized to 15 wt% to ensure a satisfying stability of BP in a wide temperature range as well as a preferable EO tunability, *i.e*., Kerr effect; while the content of photoinitiator was 0.4 wt% to promote the polymerization of monomers. Such prepared mixture was homogeneously stirred at clearing point for about 30 minutes and then injected into a 15-μm-thick cell with inter-digital electrodes on one of substrate, *i.e*., IPS cell (width of electrode: 15 μm; gap between the adjacent electrodes: 15 μm), by capillarity. Such mixture was retained at a typical BP texture by accurately holding the temperature of sample in BP range using a precisely controlled hot stage (Instec HCS410), and followed by a light irradiation of ultra violet (UV) light-emitting diode (LED) with a central wavelength of 365 nm and an output intensity of 1.5 mW/cm^2^ for about 15 minutes.

### Sample testing

Thermal stability of such PSBPLC sample was confirmed through the optical texture observed by a polarized optical microscope (POM, Nikon LV100POL) during a heating-and-cooling cycle with a settled rate of 0.5 °C/min. The probe light generated by a double-frequency neodymium-doped yttrium aluminium garnet (Nd:YAG) laser (532 nm, 10 mW/cm^2^) impinged on the sample along the cell normal for testing EO performances. The polarization direction of the probe beam was modulated to ± 45° with respect to the orientation of IPS electrodes, to ensure a larger transmission contrast between applying and removing the driving voltage (square wave, 1 kHz) generated from a signal generator (Tektronix, AFG3022). Kerr constant of the sample was obtained by fitting the dependency between the square of applied electric-field and the field-induced birefringence of the sample in accordance with the extended Kerr equation^34^; while the birefringence was measured through the commonly used Senarmont’s method^35^. The response behaviour was monitored by a photoelectric-converter-connected oscilloscope.

## Electronic supplementary material

Supplementary Information
