# Supplementary material for: Enhanced Low-temperature Electro-optical Kerr Effect of Stable Cubic Soft Superstructure Enabled by Fluorinated Polymer Stabilization
Source: Sci Rep. 2017 Sep 4;7:10383. doi: 10.1038/s41598-017-11041-2 (PMC5583357; doi:10.1038/s41598-017-11041-2)
Supplement: Supplementary file 1 — Supplementary Information [file 41598_2017_11041_MOESM1_ESM.pdf]

Supplementary Information

Enhanced Low-temperature Electro-optical Kerr Effect of Stable Cubic Soft Superstructure Enabled by Fluorinated Polymer Stabilization

Xiao Li[1,2], Wei-Qiang Yang[1], Cong-Long Yuan[1], Zhen Liu[1], Kang Zhou[1,2], Xiao-Qian Wang[1], Dong Shen[1], Zhi-gang Zheng[1,*]

[1]Physics Department, East China University of Science and Technology, Shanghai, 200237, China.
[2]School of Materials Science and Engineering, East China University of Science and Technology, Shanghai, 200237, China.
[*]E-mail: zgzheng@ecust.edu.cn

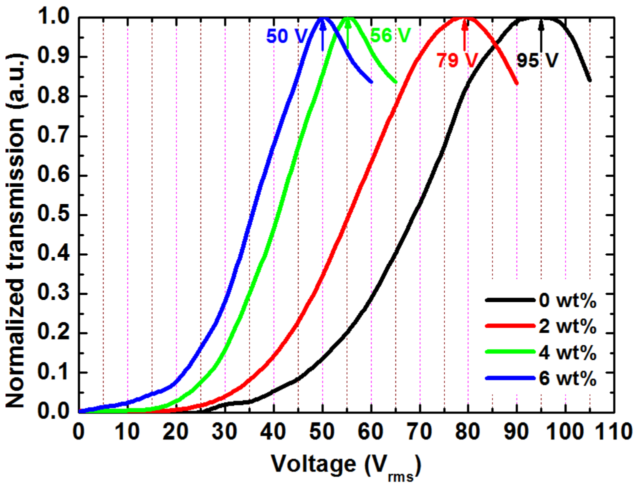

Figure S1. Voltage-dependent-transmission performances of PSBPLCs with an enhanced fluorination at the room temperature. The driving voltage, labelled on each curve, is defined as the voltage corresponds to the maximum transmission (~20 ^{o}C).

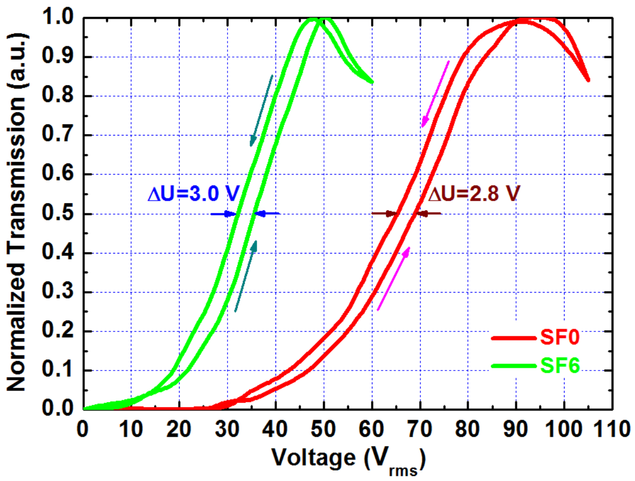

Figure S2. Voltage-dependent-transmission performances of the non-fluorinated (SF0, red curves) and fluorinated (SF6, green curves) samples tested in a rising-and-reducing loop of the applied voltage at the room temperature (~20 ^{o}C).

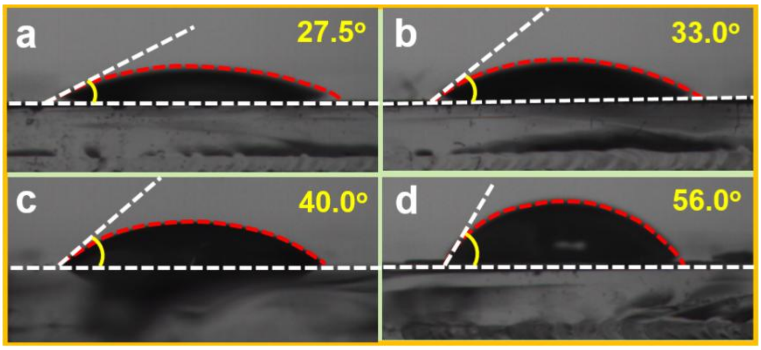

Figure S3. Contact angle of LC TEB300 on the surface of polymer film formed by (a) non-fluorinated, (b) 2 wt%-fluorinated, (c) 4 wt%-fluorinated and (d) 6 wt%-fluorinated monomers. The corresponding contact angles were labelled at the top right corner of the panels.

Molecular dynamics simulation

Molecular dynamic (MD) simulations were implemented as the following three steps: firstly, some amounts of molecules were settled into a cubic box with a certain dimensions determined by the density of material. Such cubic box always brings many rebounds, which was inexistent in the real system, due to the colliding of molecules with the walls of the box, thereby changing the moving direction of the molecules during MD process. Therefore, the periodic boundary condition was necessary to be set before the simulations to avoid the influences of the walls in the simulations.[1,2] Subsequently, an appropriate force field was selected to move every

atom contained in the molecules reaching the most stable (*i.e.*, the lowest energy) position. The force field, a potential energy function of the system, is normally expressed by the coordinates of the centre-of-mass, thereby deducing the acceleration, the velocity and the displacement of the atoms by Newton's law.[3] At the initiation, an initial velocity was generated and assigned to every atom, and simultaneously the total momentum of the system was ensured to be zero; thus these atoms would be restrained by the force field and moved to the next certain position. Such process was repeated until the entire system reaches thermal equilibrium. Thirdly, a proper statistical ensemble algorithm is adopted to conduct the thermodynamic relaxation at a defined temperature.

Herein, the extent of fluorination was simulated by changing the molar concentration of fluorine in the model in accordance with the real molar concentration in the experiment. The interface was generated by stacking a LC layer on a fluorinated layer with the restriction of the atomic interactions, i.e., the bilayer model. Thermo-equilibrium of the bilayer system was performed and restrained by the polymer consistent force field (PCFF). By means of the obtained coordinates from the simulations, the bond length and angle, the out-of-plane dihedral angle, as well as the correlations between them were calculated through the mathematic expression of PCFF, and thereby the bond energy of the system was obtained, which was the intramolecular action; similarly, the non-bond energy, such as static electronic interaction and the Van der Waals interaction, which were directly determined by the distance between two molecules, was calculated; and the sum of these items of bond and non-bond energies was the total energy of the system. Provided that the total energy of the system was denoted as E_0; the energies of LC layer and fluorinated layer were E_{LC} and E^*, respectively; therefore, the interaction energy on the interface, E_{int}, was deduced as $E_{int}=E_0-(E_{LC}+E^*)$.[4]

1. Z. Zheng, J. Ma, X. Li, *J. Phys. D Appl. Phys.* **41**, 235302, (2008).

2. N. Metropolis, A. W. Rosenbluth, M. N. Rosenbluth and A. H. Teller, *J. Chem. Phys.* **21**, 1087, (1953).
3. J. A. Fay, Molecular Thermodynamics, *Addison-Wesley Publishing Company*, (1965).
4. S. S. Patnaik and R. Pachter, *Polymer* **40,** 6507, (1999).
